# Signalling Network Analysis of Blood Mononuclear Cells From Clinical Samples by Bivariate Correlation

**DOI:** 10.1111/jcmm.70550

**Published:** 2025-06-20

**Authors:** David Kaplan, Hillard M. Lazarus, Jason Valent, Faiz Anwer, Sandra Mazzoni, Christy Samaras, Louis Williams, Megan Nakashima, Mazen Hanna, Shahzad Raza, Eric Christian, Jack Khouri

**Affiliations:** ^1^ CellPrint Biotechnology LLC Shaker Heights Ohio USA; ^2^ Department of Medicine Case Western Reserve University Shaker Heights Ohio USA; ^3^ Department of Hematology and Medical Oncology, Taussig Cancer Center Cleveland Clinic Foundation Cleveland Ohio USA; ^4^ Department of Pathology and Laboratory Medicine Cleveland Clinic Foundation Cleveland Ohio USA

**Keywords:** AL amyloidosis, apoptosis, MGUS, myeloma, peripheral blood mononuclear cells, phosphoantigens, plasma cell dyscrasia, signalling pathways, STING pathway

## Abstract

Signalling networks have been assessed in blood cells by assessing individual phosphoantigens. We considered the possibility that bivariate correlations involving a series of signalling molecules could be used to delineate functional signalling networks in cells from clinical samples. Here, we describe a novel approach to signalling network analysis using enhanced flow cytometry to provide increased resolving power and restricted‐dimensional cytometry which simplifies the analysis so that the precision of the analysis is optimised. This approach has been validated in short‐term cultures by recapitulating known tenets of two distinct pathways. Additionally, new findings from our unique approach provide both expanded and nuanced views of signalling circuits. Applying our technology platform to blood mononuclear cells from patients with plasma cell disorders, we identified cell‐type specific features of signalling pathways by distinct patterns of bivariate correlations. The intermolecular relationships between signalling analytes provide a description of the signalling network in blood cells from clinical samples. Consequently, our approach has the potential to assess how the blood mononuclear cell‐type specific signalling network affects pathophysiology and pathogenesis.

## 1 Introduction

Intracellular signalling pathways have been assessed in blood cells from clinical samples by measuring the levels of phosphoantigens [1]. Consequently, increased levels of phosphorylated Akt indicate that the PI3K/Akt pathway is activated [2], or enhanced levels of phospho‐RelA are evidence of activity in the NFκB pathway [3, 4]. However, most phosphorylated substrates are not involved in single pathways, but instead operate in multiple pathways, forming more of a network than a series of discrete, independent tracks [1, 4, 5]. For instance, GSK3 is a kinase implicated in various pathways involving diverse cellular functions such as apoptosis, cell survival, protein translation and cell cycle progression [6].

We considered the possibility that the signalling network may be delineated in blood mononuclear cells from clinical samples by assessing bivariate correlations of informative analytes. To achieve this goal, we used an enhanced flow cytometric technology that allows for 10–100‐fold greater resolving power than standard flow cytometry [7, 8, 9, 10, 11, 12, 13, 14, 15, 16, 17, 18, 19, 20, 21, 22, 23] and restricted‐dimensional cytometry (rdC) that simplified the analysis [7]. The signal amplification technology enhanced the resolving power of the analysis so that low‐abundance analytes could be detected quantitatively, and rdC demonstrated exceptional precision so that bivariate correlations with high levels of *r* could be obtained. Our technology platform was used to ascertain the relationships among selected signalling analytes, and thereby we were able to delineate the signalling network in mononuclear blood cells from clinical samples.

## 2 Methods

### 2.1 Cell Donors

Mononuclear cells were obtained from healthy volunteers at the Comprehensive Cancer Center core facility of Case Western Reserve University. Patients diagnosed with various plasma cell dyscrasias (myeloma, MGUS, AL amyloidosis) according to IMWG criteria were managed at the Cleveland Clinic Foundation Taussig Cancer Center. The myeloma and AL amyloidosis patients were either newly diagnosed or on various plasma cell‐directed therapies. Blood was obtained between 9:00 AM and 1:00 PM and processed within 4 h of venipuncture.

### 2.2 Study Approval

All blood donations from healthy volunteers were approved by the Institutional Review Board at Case Western Reserve University, and informed consent was obtained prior to participation. The study involving patient samples was conducted according to the guidelines of the Declaration of Helsinki and approved by the Institutional Review Board of the Cleveland Clinic Foundation. Informed consent was obtained from all patients prior to participation.

### 2.3 Cells

Discontinuous gradient centrifugation was used to isolate mononuclear cells in blood samples from donors. The isolated mononuclear cells were viably frozen in 10% dimethyl sulfoxide and stored in liquid nitrogen until use.

### 2.4 Cell Culture

For the study of ABT263 effects, mononuclear cells frozen in dimethyl sulfoxide were thawed and then cultured at 1 million cells per mL overnight in RPMI 1640 with 10% fetal bovine serum at 5% CO_2_ at 37°C. The cultures included 1 μM ABT263.

### 2.5 Reagents

ABT263 was obtained from Seleckchem. For the study of the STING pathway, diABZI was obtained from Cayman Chemical. Primary antibody reagents were obtained from commercial sources. For the study involving cultured cells, antibodies with specificities for Mcl1 (cat#32087), PUMA (cat#33906), Bad (cat#32445), Bid (cat#32060), Bak (cat#32371), PTEN (cat#133254), Bcl2A1 (cat#33862), Bcl2 (cat#32124), Shp1 (cat#124942), Atg7 (cat#52472), phospho‐Lyn (cat#40660), WT1 (cat#89901), Musashi2 (cat#76148) and ZAP70 (cat#32429) were obtained from Abcam. Antibodies with specificities for survivin (cat#2808), BclxL (cat#2764), Bim (cat#2819), phospho‐RelA ser536 (cat#3033), phospho‐GSK3β ser9 (cat#9323), phospho‐Akt thr308 (cat#2965), phospho‐Erk thr202/tyr204 (cat#9101), phospho‐ZAP70 tyr493/phospho‐Syk tyr526 (cat#2704) and phospho‐Bcl2 ser70 (cat#2827) were obtained from Cell Signaling Technology. An antibody specific for Bax (cat#PA5‐11378) was obtained from Thermo. An antibody specific for SMN (cat#FCMAB153F) was obtained from Millipore. An antibody specific for Bcl2L12 (cat#3745‐1) was obtained from Epitomics. For the study involving plasma cell dyscrasia patients, antibodies with specificities for BDNF (cat#108319), calmodulin (cat#45689), Vav (cat#40875) and HMGB1 (cat#79823) were obtained from Abcam. Antibodies with specificities for phospho‐Bcl2 ser70 (cat#2827), phospho‐cJun ser73 (cat#3270), phospho‐Erk thr202/tyr204 (cat#4370), phospho‐GSK3β ser9 (cat#5558), phospho‐p38 MAPK thr180/tyr182 (cat#9215), phospho‐RelA ser536 (cat#3033), phospho‐RIP ser166 (cat#44590), phospho‐Shp2 tyr580 (cat#5431), phospho‐sequestasome ser349 (cat#16177), phospho‐STING ser366 (cat#cat#40818), phospho‐TBK1 ser172 (cat#5483), phospho‐ULK1 ser757 (cat#14202), phospho‐ZAP70 tyr319/phospho‐Syk tyr352 (cat#2717) and Syk (cat#13198) were obtained from Cell Signaling Technology. An antibody with specificity for HO‐1 (cat#M00253) was obtained from Boster. For the study of the STING pathway, antibodies with specificities for phospho‐STING ser366 (cat#5431), phospho‐TBK1 ser172 (cat#2717), phospho‐RelA ser536 (cat#3033) and phospho‐IRF3 ser396 (cat#29047) were obtained from Cell Signaling Technology.

### 2.6 Enhanced Flow Cytometric Analysis

Cells were thawed, stained for the expression of lineage markers (CD4 for CD4^+^ T cells and monocytes, CD8 for CD8^+^ T cells and CD19 for B cells) in separate tubes, fixed in the presence of paraformaldehyde and permeabilised with saponin. Labelled co‐staining antibodies were obtained from BioLegend (San Diego, CA). Because many of the analytes of functional interest are expressed in low abundance, we amplified the specific signals by a powerful catalysed reporter deposition method as previously described [7, 8, 9, 10, 11, 12, 13, 14, 15, 16, 17, 18, 19, 20, 21, 22, 23]. Briefly, the cells were sequentially incubated at room temperature with primary antibodies and then either anti‐rabbit or anti‐fluorescein antibodies conjugated with horseradish peroxidase. The cells were then exposed to hydrogen peroxide and fluoresceinated tyramide at room temperature. The cells in each tube included a single lineage marker and antibodies to a single pathway analyte whose signal was amplified. A BD Accuri flow cytometer was used to analyse the cells, and the results were analysed using FloJo software. The average number of events per peak was 6246.

Flow cytometric data collection was performed with the technologists unaware of the diagnosis associated with the de‐identified samples, of any other clinical data and of the purpose of the study.

### 2.7 Statistical Analysis

ANOVA, *t*‐tests and correlational matrices were calculated with SPSS and Excel software. The significance of differences between two correlation coefficients was calculated online at vassarstats.net/rdiff.html.

## 3 Results

### 3.1 Quantitation of Molecular Expression Levels in a Cellular Signalling Pathway

**FIGURE 1:**
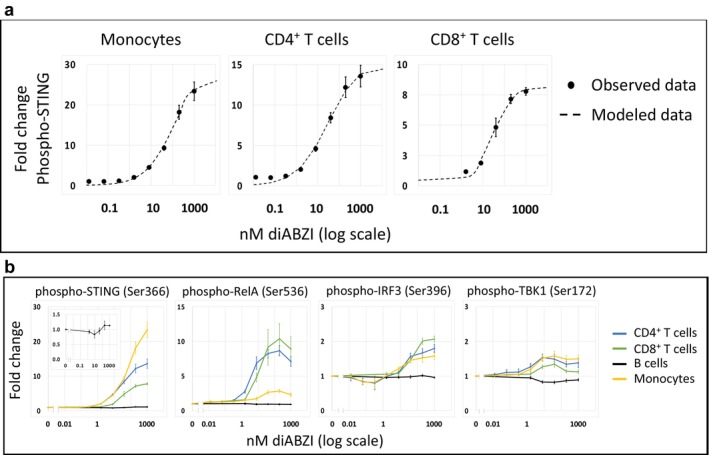
STING agonist initiates the STING pathway. (a) Peripheral blood mononuclear cells from six healthy volunteers were treated with varying concentrations of the STING agonist diABZI. Expression levels of phospho‐STING were assessed in T cells and monocytes by signal amplification and rdC. Error bars represent standard errors. Modelled data (dashed lines) show the expression levels calculated by the Hill equation. (b) Peripheral blood mononuclear cells from six healthy volunteers were treated with varying concentrations of the STING agonist diABZI for 1 h. Expression levels of various phosphoantigens in the STING pathway were assessed in four cell types. Fold changes in expression levels of the various phospho‐antigens are plotted against the STING agonist concentration on a log scale. In the left panel, an inset shows the expression of B cell phospho‐STING at a lower level of fold change. Error bars represent standard errors. The inset in the left panel shows the results in B cells plotted on a different scale.

To determine if flow cytometry with enhanced resolving power and restricted dimensionality could assess a well‐described signalling pathway with precise quantitation, we cultured mononuclear cells from six healthy donors with varying concentrations of the STING agonist di‐amidobenzimidazole (diABZI) [24] for 1 h, and the expression of phosphorylated STING was assessed (Figure 1a). Data for CD4^+^ T cells, CD8^+^ T cells and monocytes show agonist‐dependent STING activation in a way that conforms to the Hill equation.

Downstream consequences of STING activation include various phosphorylated substrates such as TBK1, IRF3 and RelA [25, 26]. We tested for the expression of these phosphorylated substrates in CD4^+^ T cells, CD8^+^ T cells, B lymphocytes and monocytes (Figure 1b). These components of the STING pathway were stimulated in a dose‐dependent manner, illustrating the capacity of our technology platform to assess signalling pathway activation in a quantitative way.

### 3.2 Bivariate Correlations of Integral Proteins Linked to a Specific Intracellular Pathway

We considered the possibility that bivariate correlations obtained by our approach could be used to assess the signalling network. For instance, an increase in phospho‐Akt induces an increase in phospho‐mTor which, we postulate, may be seen as a high level of correlation between these two analytes.

To determine if we could identify signal networks by bivariate correlations, we studied signalling and apoptotic pathways in B lymphocytes among peripheral blood mononuclear cells cultured overnight in the presence of ABT263 (navitoclax), a Bcl2/BclxL/Bclw inhibitor. In this study, we analysed results from 31 healthy volunteers, which is adequate to ensure the statistical significance of bivariate correlations. ABT263 was added at a concentration (1 μM) which did not induce apoptosis overnight but did induce cell death by 2 days of culture.

**FIGURE 2:**
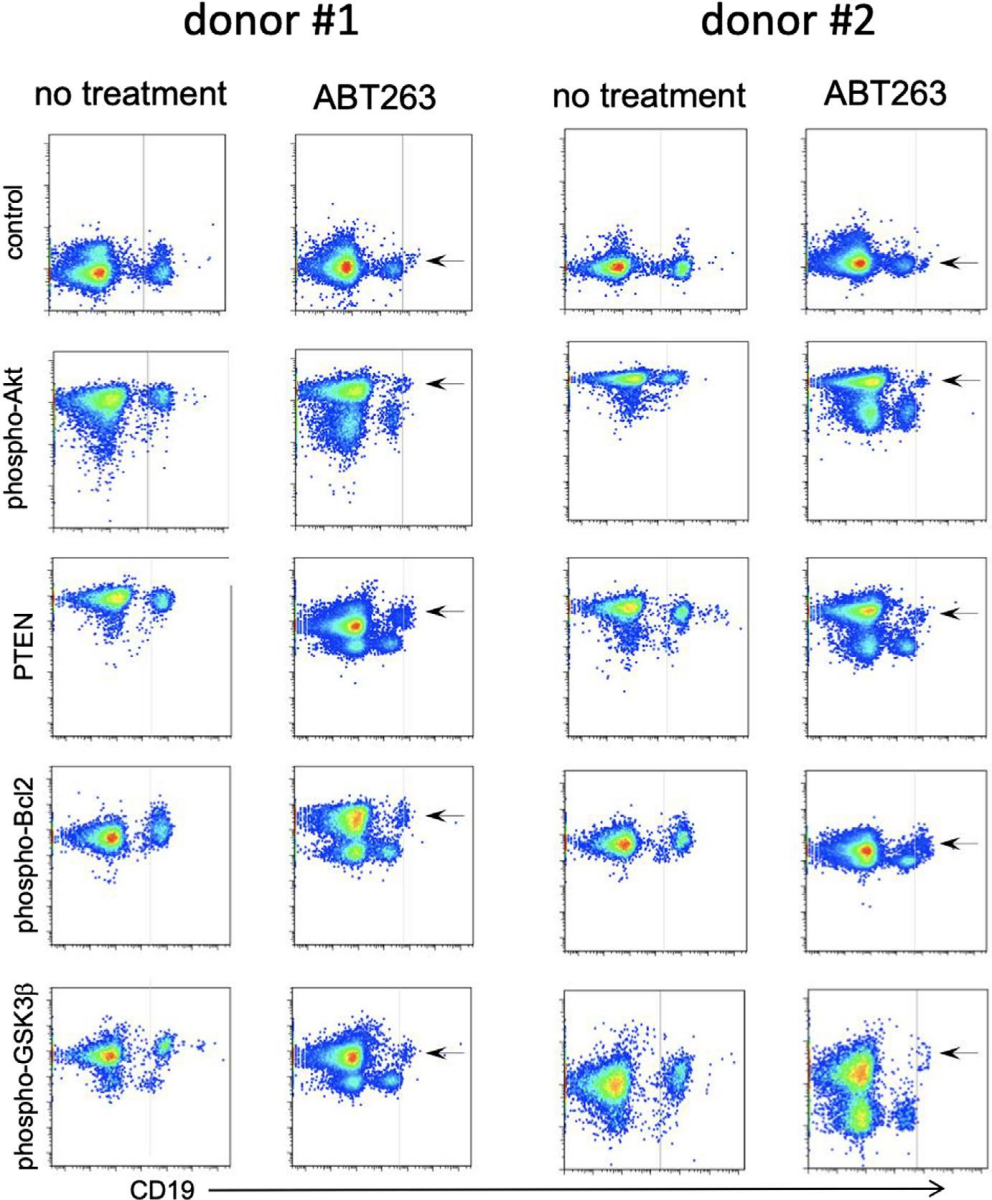
Dot blots of representative flow cytometric analysis. Various analyte expression levels are shown on the *y*‐axis, and CD19, a marker for B cell lineage, is shown on the *x*‐axis. The analyte expression levels were amplified, and rdC was used to enhance the precision of the analysis. The vertical cursor in each panel is placed to show the decrease in CD19 expression after ABT263 treatment. Results from two distinct donors are shown, with the left two columns and the right two columns showing results from different donors. Arrows identify the rare subpopulation of B cells that appear only in the samples cultured with ABT263.

Representative data for four analytes and control and two distinct donors are shown in Figure 2. These data demonstrate the clarity of the original data obtained in our studies. The splitting of B cells into a major population and rare subpopulation induced by the presence of ABT263 is shown here. The distinction of these groups was facilitated by the differential effects on both the analytes in the signalling or apoptotic pathways and by the distinct levels of CD19 expression, which was used to identify B cells.

**FIGURE 3:**
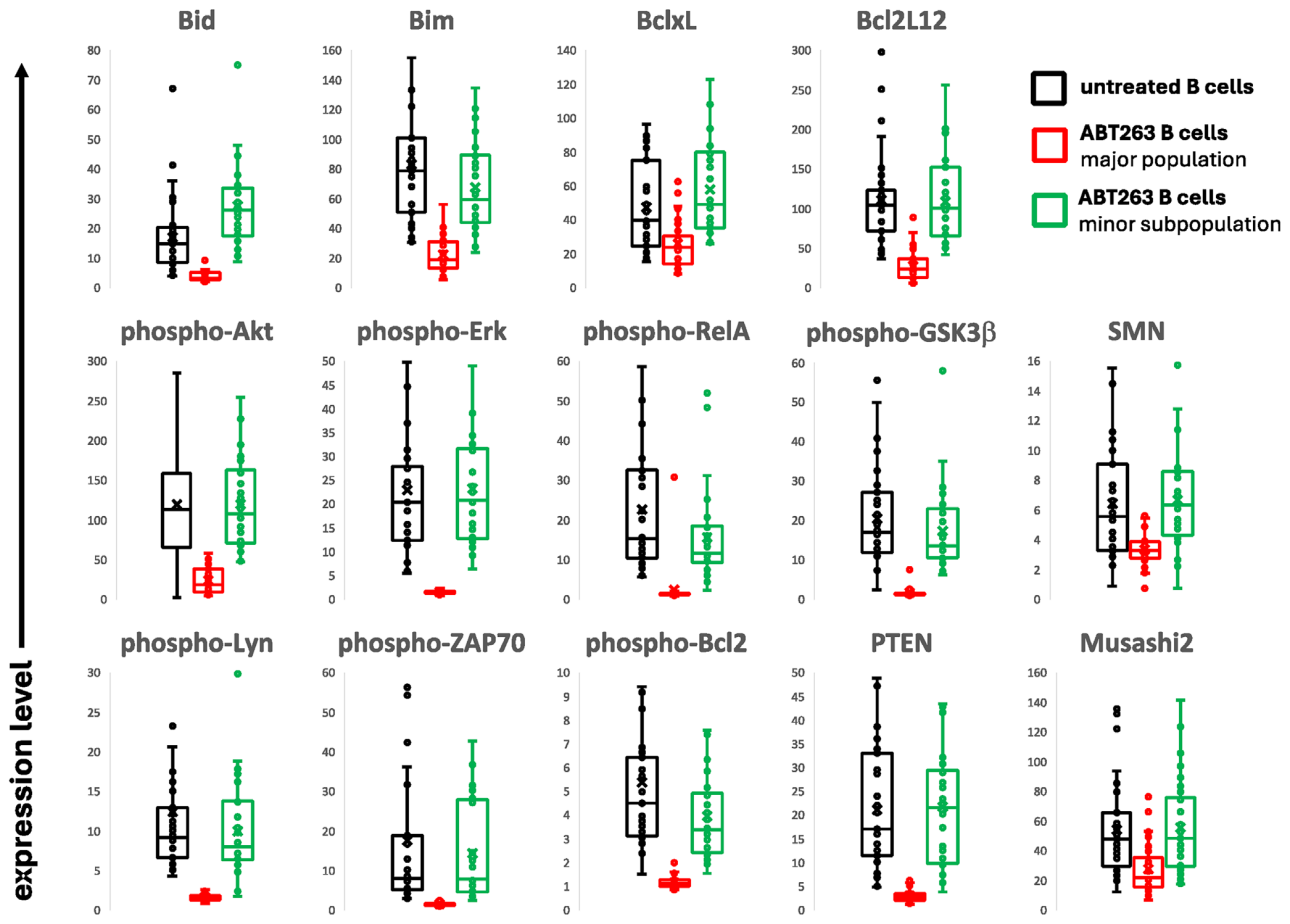
Effects of ABT263 on B cell analyte expression levels. PBMC were cultured overnight in the presence of ABT263, a Bcl2/BclxL/Bclw inhibitor and expression levels for the indicated analytes were obtained from 31 independent donors.

Twenty‐six analytes related to signalling and apoptosis were included in the study. Fourteen of the analytes showed a consistent pattern. The major population of B cells demonstrated significantly decreased analyte expression levels after exposure to ABT263, and the rare subpopulation did not (Figure 3). Statistical analysis for the data in Figure 3 is shown in Table S1. With the exception of Bid, the analyte expression level of the rare B cell subpopulation was not distinguishable from the analyte expression level of the untreated B cells.

The remaining 12 analytes did not demonstrate this pattern. Most of them exhibited no expression‐level effects in the presence of ABT263 (Figure S1). Statistical analysis for the data in Figure S1 is shown in Table S2. Bcl2 (a target of the agent), Shp1 and survivin showed an inverted pattern with increased levels in the major population after ABT263 treatment.

We noted that all seven phosphoantigens showed a marked loss of expression in the major population of treated B cells. We considered the possibility that the decrease in the expression of the phosphoantigens was related to phosphatase activity. Since we included two phosphatases in the analysis (Shp1 and PTEN), we looked to see if the level of the phosphatases was related to the loss of the phosphoantigens (Table S3). The changes in the expression levels of phospho‐Akt, phospho‐Erk, phospho‐GSK3β, and phospho‐RelA were significantly correlated with both Shp1 and PTEN levels in the untreated cells; however, the change in expression of phospho‐Lyn and phospho‐Bcl2 were not correlated with either phosphatase. The change in phospho‐ZAP70 was significantly correlated with the level of PTEN but not Shp1.

To assess the relative contribution of the two phosphatases to the change in phosphoantigen expression, we analyzed the results by multiple linear regression (Table S4). The changes in phospho‐Akt and phospho‐GSK3β were explained in a model with both Shp1 and PTEN although the effects of Shp1 were clearly dominant. The changes in phospho‐Erk and phospho‐RelA were explained by PTEN levels, but Shp1 did not significantly contribute. The regression models for changes in phospho‐Akt and phospho‐GSK3β are shown in Figure S2.

**FIGURE 4:**
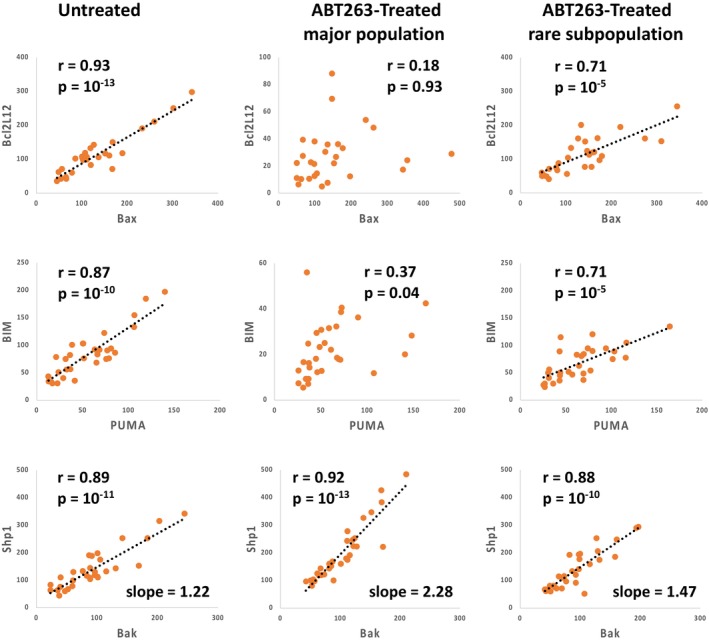
Bivariate correlations for selected molecular pairs according to cellular treatment. PBMC were untreated or cultured overnight in ABT263, and expression levels for various analytes were obtained by rdC. Bivariate correlations were calculated, and the associated *p* values are shown. Linear regressions are shown as dotted lines.

Bivariate correlations provided insights into mechanisms of dephosphorylation induced as a part of the apoptotic process. They also have the potential to reveal the intermolecular organization of cells. We observed significant changes in bivariate correlations based on exposure to ABT263. For instance, Bcl2L12 and Bax were significantly correlated in the untreated B cells and in the ABT263‐treated rare subpopulation, but did not exhibit associated expression in the ABT263‐treated major population (Figure 4). The same relationships were seen for Bim and PUMA. Shp1 and Bak were correlated in all three groups but the slopes of the regressions were different for the ABT263‐treated major population. The *p* values for the differences in r values after *r*‐to‐*z* transformation are shown in Table S5.

**FIGURE 5:**
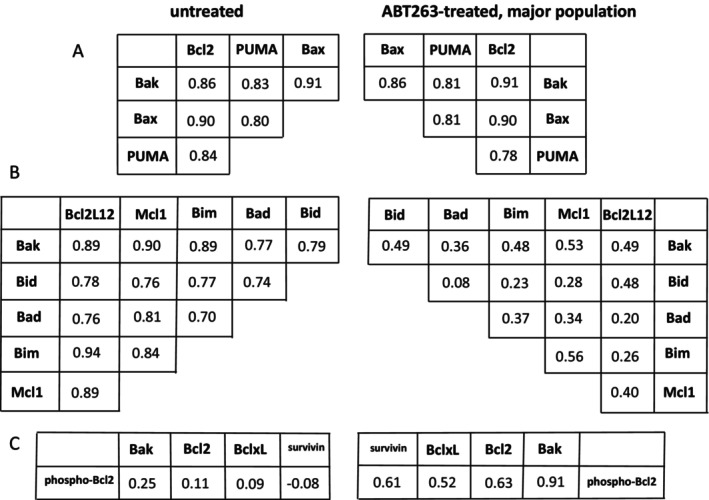
Bivariate correlations for apoptotic pathway molecules. Untreated B cells (left) and ABT263‐treated, major population B cells (right) are shown with corresponding matrices seen as mirror images. Correlation matrices show the corresponding bivariate *r* values. (A) Bivariate correlations are equivalent in both sample sources. (B) Bivariate correlations are high in the untreated samples but not in the treated samples. All differences show *p* values < 0.01 after *r*‐to‐*z* conversion. (C) Bivariate correlations are low in the untreated samples but higher in the treated samples. The *p* value for phospho‐Bcl2 versus Bcl2 after *r*‐to‐*z* conversion is 0.018 and the *p* value for phospho‐Bcl2 versus BclxL after *r*‐to‐*z* conversion is 0.068. The other relationships have *p* values < 0.01 after *r*‐to‐*z* conversion.

In our study several patterns of bivariate correlations provide novel insights into the process of apoptosis. Among the eleven analytes in the apoptotic pathway included in the analysis, four demonstrated high levels of intercorrelation that were maintained after treatment with ABT263, and six demonstrated high levels of mutual correlation in the untreated samples but not after ABT263 treatment (Figure 5A,B). Additionally, phospho‐Bcl2 was not correlated with any other analyte in the untreated samples but showed significant correlations with four other analytes after treatment with the pharmaceutical agent (Figure 5C). These results demonstrate sets of molecules that are associated with apoptotic induction in diverse ways. The significant number of samples included in the analysis allows for the use of bivariate correlations to understand how pathway molecules relate to each other.

### 3.3 Pathway analysis derived from clinical samples

Having established the technology platform on samples manipulated in culture, we sought to assess our analytic platform on patient samples. Peripheral blood samples were collected from 78 patients with various plasma cell dyscrasias including MGUS (*n* = 9), smoldering myeloma (*n* = 1), active myeloma (*n* = 41), and AL amyloidosis (*n* = 27). The different pathological processes were included in order to generate clinically relevant variance. We assessed expression levels for 20 analytes in two cell‐types, CD4^+^ T cells and monocytes. The analytes were chosen on the basis of their potential involvement in the amyloidosis disease process. We posited that tissue amyloid deposits may interact with receptors on CD4^+^ T cells and monocytes and result in changes in detectable pathway molecule expression levels.

**FIGURE 6:**
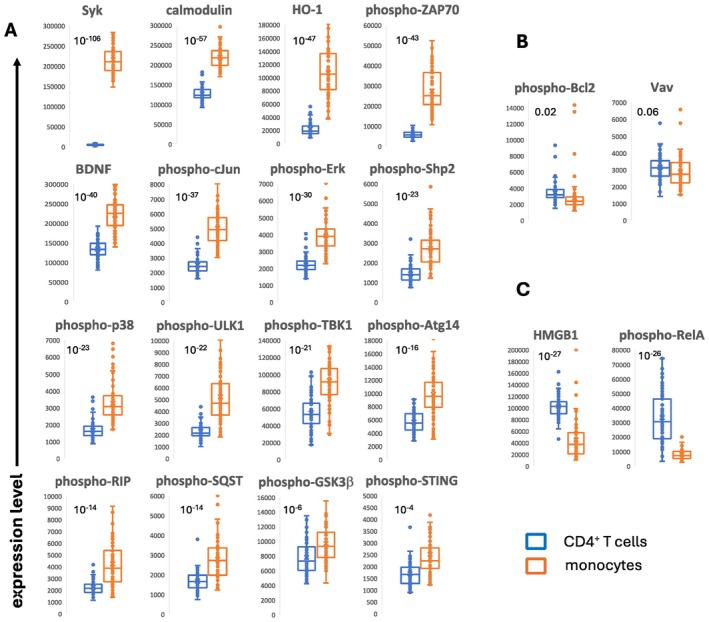
Expression levels of analytes in CD4^+^ T cells and monocytes from patients with plasma cell dyscrasias. Peripheral blood mononuclear cells were obtained from patients and were assessed for analyte expression levels. The *p* values for the comparison between expression levels in the two cell‐types is shown in each panel in the upper left corner. (A) Analytes that expressed higher amounts in monocytes compared to the lymphocytes are shown. (B) Analytes that expressed approximately the same level in the two cell‐types are shown. (C) Analytes that expressed higher amounts in the lymphocytes compared to the monocytes are shown.

Figure 6 shows expression levels for both cell‐types and comparisons for the relative expression between the cell‐types. Most of the analytes were expressed at higher levels in the monocytes (Figure 6A) which may be a reflection in part of the larger size of monocytes compared to lymphocytes [27]. Phospho‐Bcl2 and Vav were expressed in approximately the same level in the two cell‐types although both the molecules were slightly more abundant in CD4^+^ T cells than monocytes (Figure 6B). For HMGB1 and phospho‐RelA, expression in the lymphocytes was clearly greater than in the monocytes (Figure 6C).

**FIGURE 7:**
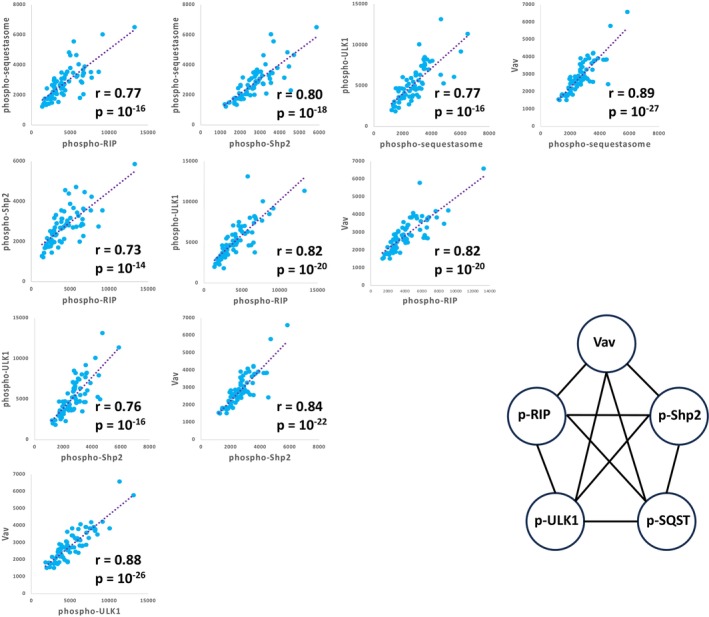
Bivariate correlations associated with monocytes from plasma cell dyscrasia patients ex vivo. Bivariate relationships are shown with linear regressions. Both *r* values and their associated *p* values are shown in the lower right corner of each panel.

The correlation matrix was calculated for both cell‐types (Figure S3), and it revealed various associations identified by high levels of bivariate correlations. First, Vav, phospho‐RIP, phospho‐Shp2, phospho‐ULK1, and phospho‐sequestasome were found to form a highly intercorrelated set of molecules in monocytes (Figure 7). These same molecules were also related in CD4^+^ T cells although the correlations were not as strong (Figure S3). Second, the phosphoantigens of the MAPK pathway (phospho‐Erk, phospho‐p38, phospho‐cJun) were significantly correlated with each other and with phospho‐Shp2 (Figure S4) in CD4^+^ T cells. A less impressive circuit of these pathway molecules was also seen in monocytes but without including phospho‐cJun (Figure S3). Finally, in monocytes BDNF, calmodulin, and Syk were highly intercorrelated (Figure S5). In CD4^+^ T cells BDNF and calmodulin were correlated (*r* = 0.82; *p* = 10^−20^), but Syk, which is expressed in low abundance in CD4^+^ T cells (Figure 3), was not correlated (Figure S3).

## 4 Discussion

Assessing signalling networks for their involvement in pathophysiology and pathogenesis is an important goal for biomedical scientists. An analysis of signalling pathways in clinical samples by bivariate correlations requires exceptional resolving power so that low abundant analytes can be detected and exceptional precision so that the relationship between multiple analytes can be visualised. Our amplification technology [7, 8, 9, 10, 11, 12, 13, 14, 15, 16, 17, 18, 19, 20, 21, 22, 23] has demonstrated excellent sensitivity and, with our recent development of rdC [7], we have achieved a high level of precision. We have used our technology platform to delineate signalling networks in blood cells from clinical samples, both confirming previously identified intermolecular relationships and discovering novel associations.

The rdC platform was established in a study of cell‐type specific expression levels of five prototypic analytes in samples from various donor groups [7]. Blood mononuclear cell‐type specific expression of the five analytes distinguished pregnant from nonpregnant women and G‐CSF‐treated from untreated persons. Also, the analysis was precise enough to demonstrate a novel relationship between RelA and translocator protein by correlational analysis. This capability prompted us to consider whether bivariate correlations detected by rdC [7] could be useful in assessing signalling networks in samples from patients.

Although rdC was first enunciated in these demonstrations [7], the antecedents to this technology have been previously revealed over many other studies [10, 12, 15, 18, 23]. The consistent characteristics in all of these investigations are the exceptional sensitivity and precision of the analysis.

In this study, we first established that our platform was capable of delineating signalling pathways in two experimental systems that involved in vitro manipulations. Then, we studied clinical samples from patients with various plasma cell dyscrasias and found bivariate relationships that revealed the configuration of signalling in specific cell types of blood mononuclear cells.

In the first experimental system, our analysis of the STING pathway in peripheral blood mononuclear cell types included B and T lymphocytes and monocytes. We demonstrated the capacity of our technology to delineate a signalling pathway, although our sample size did not allow for a clear assessment of the potential role of bivariate correlation.

We did not assess the STING pathway in dendritic cells or NK cells, which would have been reasonable considering their abundant production of class I interferon. We included lymphocytes in our analysis because of their relative abundance in the peripheral blood. The average number of cells represented in each peak was > 6000. To obtain that number, we limited our analysis to the cell types that are more plentiful. We believe that the analysis of more cells enhances the precision of our analysis, although we have not yet rigorously investigated this assumption.

Our approach focuses on precise relative quantitative analysis of cell‐type specific molecular expression levels [7]. This attribute is demonstrated by the bivariate plots. An advantage of precise relative quantitative analysis is the visualisation of bivariate relationships over a range of expression levels that actually occur in the clinical samples. Consequently, it involves ranges of expression levels that are relevant.

In the present study, the bivariate relationships were linear; however, in other studies, we have seen both exponential and logarithmic associations [15, 18]. Although the interpretations of the various relationships are not clear at this time, further investigations will enhance our understanding of the meaning of distinct bivariate associations.

Bivariate relationships were used to delineate the signalling network in the clinical samples. However, prior knowledge of the network is clearly requisite for our approach. Bivariate relationships by themselves cannot define signalling pathways de novo since correlation does not connote causation. For instance, we cannot determine by our analysis that Vav mediates the activation of cJun or that cJun mediates the transcription of Vav or that some other factor affects both Vav and phospho‐cJun. However, prior information in the literature provides us with that information [28]. Integrating previous experimental studies with our observational analysis gives us the most complete and most powerful picture of signalling pathways in clinical samples.

An important attribute of our analysis involves attaining high levels of bivariate correlations in our studies. Higher correlation coefficients give more confidence in the results, and confidence increases exponentially so that an r value of 0.8 gives more than twice as much confidence as an r value of 0.4. With 78 subjects in our study of plasma cell dyscrasias, even low levels of correlation reach statistical significance, but low levels of statistically significant correlation do not necessarily signify biological relevance.

In the study of the effects of the apoptosis‐inducing agent, ABT263, we included enough samples to obtain correlation matrices with highly significant coefficients. The analysis of specific phosphatases and the change in phosphoantigen expression demonstrates the potential of this approach. Our results neither confirm the role of Shp1 in the dephosphorylation of Akt or GSK3β nor the role of PTEN in the dephosphorylation of Erk and RelA. Additionally, our results do not indicate that Akt and/or GSK3β are substrates for Shp1 or that Erk and/or RelA are substrates for PTEN. The associations of dephosphorylation with levels of phosphatases may not be direct. Although our investigation uncovered these novel associations, additional experimental studies are needed to understand the mechanisms of these relationships.

A close examination of the apoptotic pathway molecules in our study revealed definitive patterns of association. Expression levels of Bcl2, PUMA, Bax and Bak were highly intercorrelated before and after treatment with ABT263. This stoichiometry is particularly interesting because both Bcl2, an anti‐apoptotic protein, and PUMA, a pro‐apoptotic protein, interact with Bax and Bak, both pro‐apoptotic, to regulate apoptosis [29]. Instead of simple interactions of molecular pairs, our results suggest a higher order of organisation with all four molecules forming an interacting set.

Similarly, the six Bcl2 family members that were intercorrelated in untreated B cells but not in ABT263‐treated B cells are known to interact in a way integral to their function [29]. Mcl1 has been shown to bind to Bid, Bim, Bad and Bak, but interactions of these proteins with Bcl2L12 have not been previously reported. Furthermore, the induction of phospho‐Bcl2 correlation with several other apoptotic pathway molecules by treatment with a pharmaceutical agent was not anticipated by a review of the literature. Although Bcl2 phosphorylation is thought to regulate Bcl2 activity [30], the mechanics of this regulation have not been clearly described. An advantage of the rdC technology platform is the generation of hypotheses. The unexpected neo‐correlation of phospho‐Bcl2 with other apoptotic molecules after treatment with a Bcl2 inhibitor provides a valuable clue for understanding the mechanism of apoptosis regulation by phosphorylated Bcl2.

In our study of samples from patients with plasma cell dyscrasias, we found little expression of Syk in CD4^+^ T cells but abundant levels in monocytes. This finding is consonant with the known cellular distribution of Syk mRNA [31]. However, the greater expression of HMGB1 in the T lymphocytes compared to the monocytes was not anticipated by mRNA levels, which shows comparable amounts in the two cell types [32]. The expression levels of cell‐associated molecules, selected by their functional characteristics and distinguished by their cell type, represent a powerful modality for understanding how cells work to mediate clinical phenomena.

We found that certain signalling pathways were resolved by considering correlations among sets of molecules. The intercorrelated set in monocytes including Vav, phospho‐RIP, phospho‐Shp2, phospho‐ULK1 and phospho‐sequestasome is suggested from experimental manipulations in the literature [28, 33, 34, 35], but has not been previously demonstrated by bivariate correlations of a continuous variable over a significant detection range on clinical samples. With the exception of Vav, all of these analytes have been previously associated with autophagy [36, 37, 38, 39].

Similarly, the association of BDNF and calmodulin is presaged by the known interaction of BDNF and calcium/calmodulin‐dependent protein kinase, [40] although this understanding was achieved through studies of rat brains from animals treated with pharmaceutical agents. The possibility that Syk is also involved with these molecules has not been previously proposed.

The finding that phospho‐Erk, phospho‐p38 and phospho‐cJun are highly intercorrelated in CD4^+^ T cells suggests that the three branches of the MAPK signalling pathway are co‐regulated. The inclusion of phospho‐Shp2 in this set of molecules demonstrates the strong association of an important phosphatase in the regulation of MAPK signalling. Shp2's involvement in MAPK activity has been previously demonstrated in a study of transfected Shp2 variants in cancer cell lines [41].

## 5 Conclusions

Assessing the role of the signalling network in blood mononuclear cells to enhance understanding of pathogenesis addresses a significant unmet need. Many disorders involve alterations in signalling pathways in mononuclear cells. For instance, changes in signalling pathways have been documented in mononuclear cells for autoimmunity, cancer, infectious diseases and disorders involving inflammation. Since mononuclear cells circulate throughout the body, alterations of cell‐type specific molecular expression levels are likely to be found in the blood. We have described a simplified technology, based on a signal amplification technology and rdC, that allows for the analysis of the signalling network in blood mononuclear cells from clinical samples. This analysis is likely to provide valuable novel insights into the participation of the signalling network in pathophysiology and pathogenesis.

## Author Contributions

**David Kaplan:** conceptualization (lead), data curation (lead), formal analysis (lead), funding acquisition (lead), investigation (lead), methodology (lead), project administration (lead), resources (lead), supervision (lead), validation (lead), writing – original draft (lead), writing – review and editing (lead). **Hillard M. Lazarus:** funding acquisition (supporting), writing – review and editing (supporting). **Jason Valent:** resources (supporting), writing – review and editing (supporting). **Faiz Anwer:** resources (supporting), writing – review and editing (supporting). **Sandra Mazzoni:** resources (supporting), writing – review and editing (supporting). **Christy Samaras:** resources (supporting), writing – review and editing (supporting). **Louis Williams:** resources (supporting), writing – review and editing (supporting). **Megan Nakashima:** resources (supporting), writing – review and editing (supporting). **Mazen Hanna:** resources (supporting), writing – review and editing (supporting). **Shahzad Raza:** resources (supporting), writing – review and editing (supporting). **Eric Christian:** data curation (equal), formal analysis (equal), investigation (equal), methodology (equal), supervision (equal), validation (equal), writing – review and editing (equal). **Jack Khouri:** funding acquisition (equal), resources (equal), writing – review and editing (equal).

## Conflicts of Interest

H.M.L. is a paid consultant to Partner Therapeutics, Actinium Pharmaceuticals, CSL Behring, GlycoMimetics Inc., Jazz Pharmaceuticals, Pluristem Therapeutics Inc. and Seattle Genetics. H.M.L. also has an equity position in Partner Therapeutics, is on the speakers' bureau for Jazz Pharmaceuticals and Seattle Genetics, and is a member of the Data Safety Monitoring Board for BioSight and for Bristol‐Myers Squibb. The authors have declared no other conflicts of interest exist. J.K. has consulting/advisory roles with GPCR Therapeutics, Janssen, Prothena and Legend Biotech and research relationships with Prothena, Ascentage, Janssen, Karyopharm and GPCR Therapeutics.

## Supporting information


Data S1.

## Data Availability

The complete dataset for the study of samples from the ABT263 studies and from the patients with plasma cell dyscrasias is available upon request.
