# Supplementary material for: Signalling Network Analysis of Blood Mononuclear Cells From Clinical Samples by Bivariate Correlation
Source: J Cell Mol Med. 2025 Jun 20;29(12):e70550. doi: 10.1111/jcmm.70550 (PMC12179811; doi:10.1111/jcmm.70550)
Supplement: Supplementary file 1 — Data S1. [file JCMM-29-e70550-s001.docx]

Supplemental Table 1. ANOVA assessing the significance of analyte expression levels among the untreated and treated B lymphocyte populations

Comparison of the means from the results shown in figure 2 was accomplished by ANOVA with Tukey HSD for the correction of multiple analyses. p values are shown.

Supplemental Figure 1. Effects of ABT263 on B cell analyte expression levels. PBMC were cultured overnight in the presence of ABT263, a Bcl2/BclxL/Bclw inhibitor, and expression levels for the indicated analytes were obtained for 31 independent donors.

Supplemental Table 2. ANOVA assessing the significance of analyte expression levels among the untreated and treated B lymphocyte populations

Comparison of the means from the results shown in figure 4 was accomplished by ANOVA with Tukey HSD for the correction of multiple analyses. p values are shown.

Supplemental Table 3. Bivariate relationships between the change of phosphoantigens and the expression levels of phosphatases

Correlations between the change in phosphoantigen expression from untreated to the major population of B cells treated overnight with ABT263 and the phosphatases, Shp1 and PTEN, in the untreated samples. Values of the correlation coefficient r are shown with the p value in parenthesis. ∆ refers to the change in the expression level between cells untreated and ABT263-treated (major subpopulation).

Supplemental Table 4. Multiple linear regression analysis explaining the change in phosphoantigen expression by the expression levels of phosphatases

Multiple linear regression analysis explaining the change of phosphoantigen expression by the expression of specific phosphatases in the untreated samples. The p values for the inclusion of each phosphatase in the models are shown as well as the p value for the model. The r values for the regression are shown.

Supplemental Figure 2. Multiple linear regression of phosphatases explaining the change of expression for phosphoantigens. Shp1 and PTEN were included as independent variables for the change in expression of phosphoantigens cultured overnight with ABT263.

Supplemental Table 5. Differences in bivariate correlations among treated and untreated B lymphocytes

The p values for the differences in r values seen for the indicated molecular pairs between the three groups of B cells (untreated, ABT263-treated major population, and ABT263-treated rare subpopulation). Differences in r values were calculated after r-to-z transformation.

Supplemental Figure 3. Correlation matrices from plasma dyscrasia patients. The correlation matrices for CD4^+^ T cells (top) and monocytes (bottom) are shown. Green highlighting is shown for all bivariate correlations with r $\geq$0.55.

Supplemental Figure 4. Bivariate correlations associated with CD4^+^ T cells from plasma cell dyscrasia patients ex vivo. Bivariate relationships are shown with linear regressions. Both r values and their associated p values are shown in the lower right corner of each panel.

Supplemental Figure 5. Bivariate correlations associated with monocytes from plasma cell dyscrasia patients ex vivo. Bivariate relationships are shown with linear regressions. Both r values and their associated p values are shown in the lower right corner of each panel.
